# Na^+^-NQR Confers Aminoglycoside Resistance via the Regulation of l-Alanine Metabolism

**DOI:** 10.1128/mBio.02086-20

**Published:** 2020-11-17

**Authors:** Ming Jiang, Su-fang Kuang, Shi-shi Lai, Song Zhang, Jun Yang, Bo Peng, Xuan-xian Peng, Zhuang-gui Chen, Hui Li

**Affiliations:** a The Third Affiliated Hospital, Sun Yat-sen University, University City, Guangzhou, People’s Republic of China; b State Key Laboratory of Bio-Control, Sun Yat-sen University, University City, Guangzhou, People’s Republic of China; c Southern Marine Science and Engineering Guangdong Laboratory (Zhuhai), Zhuhai, People’s Republic of China; Michigan State University; Fred Hutchinson Cancer Research Center

**Keywords:** Na^+^-NQR, alanine metabolism, aspartate metabolism, glutamate metabolism, alanine, aminoglycoside antibiotics, cAMP/CRP, antibiotic resistance, metabolomics

## Abstract

Sodium-translocating NADH:quinone oxidoreductase (Na^+^-NQR) functions as a unique redox-driven sodium pump, generating membrane potential, which is related to aminoglycoside antibiotic resistance. However, whether it modulates other metabolisms to confer antibiotic resistance is unknown. The present study showed that loss of *nqrA* or *nqrF* led to differential metabolomes with elevated resistance to aminoglycoside antibiotics. Decreased alanine, aspartate, and glutamate metabolism and depressed abundance of alanine were characterized as the most impacted pathway and crucial biomarker, respectively. Further data showed that higher viability was detected in Δ*nqrA* and Δ*nqrF* mutant strains than their parent strain ATCC 33787 in the presence of gentamicin but recovered by exogenous l-alanine. It proceeds by the following events. The loss of *nqrA* or *nqrF* led to the decrease of membrane potential, ATPase activity, and then ATP and cyclic AMP (cAMP), which reduced the cAMP/CRP (cAMP receptor protein) complex. The reduced cAMP/CRP complex promoted l-alanine catabolism and inhibited l-alanine anabolism, causing reduced levels of alanine. Reduced alanine affected the expression of antiporter families Atp and Mnh genes. Our results suggest a novel mechanism by which the Na^+^-NQR system regulates antibiotic resistance via l-alanine metabolism in a cAMP/CRP complex-dependent manner.

## Abstract (precis)

The Na^+^-NQR complex functions as a unique redox-driven sodium pump, generating membrane potential directly. However, whether it mediates generation of membrane potential indirectly is unknown. The present study shows that the Na^+^-NQR complex impacts membrane potential through other antiporter families Atp and Mnh. It proceeds by ATP and then cAMP/CRP regulon, which inhibits l-alanine catabolism and promotes l-alanine anabolism. When the Na^+^-NQR complex is reduced as in antibiotic-resistant bacteria, l-alanine is depressed, which is related to the antibiotic resistance phenotypes. However, exogenous l-alanine reverts the phenotype and promotes antibiotic-mediated killing. These findings suggest a novel mechanism by which the Na^+^-NQR system regulates antibiotic resistance via l-alanine metabolism in a cAMP/CRP complex-dependent manner.

## INTRODUCTION

Within a relatively short time after the first antibiotics were introduced, bacteria began exhibiting various degrees of resistance (1). Antibiotic resistance is a global challenge that impacts all pharmaceutically used antibiotics (2, 3). In response to the emergent antibiotic resistance, novel strategies are needed to develop new antibiotics, vaccines, and bacitracin (4–9) and to enhance the effectiveness of existing antibiotics (10–13). Therefore, the understanding of antibiotic resistance mechanisms and identification of drug targets to battle these emerging antibiotic-resistant pathogens deserve priority status.

The sodium-translocating NADH-ubiquinone oxidoreductase (Na^+^-NQR) is found in the electron transport chain of several pathogenic and marine bacteria, including vibrios, functioning in a unique redox-driven sodium pump (14). It is a multisubunit (NqrA-F) membrane-embedded NADH dehydrogenase that oxidizes NADH and reduces quinone to quinol. It has been proposed that a scheme of electron transfer in Na^+^-NQR is initiated by NADH oxidation on subunit NqrF and leads to quinol formation on subunit NqrA. Moreover, recent reports have indicated that Na^+^-NQR plays a role in bacterial metabolism, motility, and resistance to antibiotics and osmotic stress (15–17). The lack of Na^+^-NQR harms cholera toxin levels via affecting either translation or secretion (18). The recent breakthrough in structural studies on Na^+^-NQR from Vibrio cholerae creates a perspective for the systematic design of inhibitors for this unique enzyme as a novel target for antibiotics (19). Therefore, further understanding of the role of Na^+^-NQR in antibiotic resistance is especially important to regulate the enzyme for combatting these antibiotic-resistant pathogens.

Recent reports have indicated that bacterial metabolomes contribute to susceptibility and resistance to antibiotics and serum-mediated killing (10, 20–24). Therefore, it is interesting to investigate whether Na^+^-NQR regulates antibiotic resistance through metabolic modulation. The present study demonstrates that loss of *nqrA* or *nqrF* leads to the decreased alanine, aspartate, and glutamate metabolism and the depressed abundance of alanine, which contributes to antibiotic resistance. The decrease of alanine is attributed to the regulation of *nqrA* and *nqrF* to l-alanine metabolism in a cyclic AMP (cAMP)/cAMP receptor protein (CRP)-dependent manner. Furthermore, l-alanine abundance is related to the membrane potential and intracellular concentration of gentamicin via regulating other antiporter families Atp and Mnh. The results are described below.

## RESULTS

### Resistance of Δ*nqrA* and Δ*nqrF* mutant strains to aminoglycoside antibiotics.

**FIG 1:**
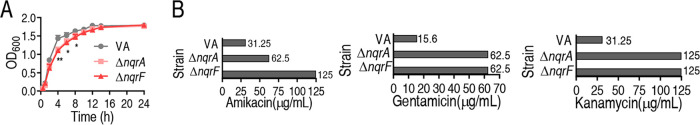
Growth and MIC of genetically modified strains with *nqrA* or *nqrF* deleted. (A) Growth curve of V. alginolyticus ATCC 33787 (VA) and Δ*nqrA* and Δ*nqrF* mutant strains. *, *P* < 0.05; **, *P* < 0.01. (B) MIC of Δ*nqrA*, Δ*nqrF*, and their parent strain (VA) to different antibiotics.

To understand the role of *nqrA* or *nqrF* in resistance to aminoglycosides, genetically modified mutants with *nqrA* or *nqrF* deleted were constructed (see Fig. S1 in the supplemental material). We first detected the growth curves of Vibrio alginolyticus ATCC 33787 and Δ*nqrA* and Δ*nqrF* mutant strains (Δ*nqrA* and Δ*nqrF*, respectively) and found that the loss of *nqrA* or *nqrF* leads to reduced growth in the exponential phase (Fig. 1A). Then, MICs of V. alginolyticus ATCC 33787, Δ*nqrA.* and Δ*nqrF* to three types of aminoglycoside antibiotics (amikacin, gentamicin, and kanamycin) were measured. The loss of *nqrA* and *nqrF* led to a fourfold elevation of MICs in medium with the three types of antibiotics except for a twofold elevation of the MIC to amikacin in Δ*nqrA* (Fig. 1B). These results indicate that the absence of *nqrA* or *nqrF* increases bacterial resistance to these antibiotics.

10.1128/mBio.02086-20.1 FIG S1 Construction of genetically modified strains with *nqrA* or *nqrF* deleted. The upstream and downstream 500-bp fragments of *nqrA* or *nqrF* were amplified (A) and then merged into a 1,000-bp fragment by overlap PCR (B). The merged fragment, which is ligated into a pRE112 vector, was identified (C) and sequenced (data not shown). Clones that grew on LB plates with 20% sucrose but not on the plates with chloramphenicol were identified by PCR using primers P4 and P7 or P7 and P8 (D and E). Meanwhile, the corresponding specific fragment using primers P5 and P6 was not amplified in the clones. These results show that *nqrA* and *nqrF* are removed from their flanking sequences through DNA recombination. (A) PCR amplification of *nqrA* and *nqrF* fragments. (B) Overlap PCR of *nqrA* and *nqrF* fragments. (C) PCR identification of *nqrA* and *nqrF* in pRE112 vector. (D and E) Identification of Δ*nqrA* (D) and Δ*nqrF* (E) by PCR. Download FIG S1, PDF file, 0.5 MB. Copyright © 2020 Jiang et al. 2020 Jiang et al. This content is distributed under the terms of the Creative Commons Attribution 4.0 International license.

### Metabolomic profiling of Δ*nqrA* and Δ*nqrF*.

Reports have indicated that bacterial metabolomes contribute to antibiotic efficacy (10, 11, 23). To explore whether the loss of *nqrA* or *nqrF* affects metabolic profiles, gas chromatography-mass spectrometry (GC-MS)-based metabolomics was used to detect metabolomes of Δ*nqrA* and Δ*nqrF*. Five biological replicates with two technical repeats in each group were performed, yielding a total of 30 data sets (Fig. S2A). This led to identification of 80 metabolites from each sample. The high reproducibility of the identification in the discovery phase is shown in Fig. S2B. The biological categories of the identified metabolites were searched for in the Kyoto Encyclopedia of Genes and Genomes (KEGG). The categories showed that 18.75% (16), 32.50% (26), 27.50% (22), and 16.25% (13) of metabolites belong to carbohydrates, amino acids, lipids, and nucleotides, respectively (Fig. S2C).

10.1128/mBio.02086-20.2 FIG S2 Metabolic profiles of Δ*nqrA* and Δ*nqrF*. (A) Unsupervised hierarchical clustering of all metabolites (row). Yellow and blue indicate an increase and decrease of metabolites relative to the mean and standard deviation of the row metabolite level, respectively (see color scale). Vibrio alginolyticus VA (ATCC 33787), Δ*nqrA*, and Δ*nqrF* labels at the top indicate the category groupings. (B) Reproducibility of the metabolomics profiling platform used in the discovery phase. Metabolite abundances quantified in samples over two technical replicates are shown. The correlation coefficient between technical replicates varies between 0.998 and 0.999. This plot shows the two replicates with the weakest correlation of 0.998. (C) Categories of detected metabolites. Download FIG S2, TIF file, 2.5 MB. Copyright © 2020 Jiang et al. 2020 Jiang et al. This content is distributed under the terms of the Creative Commons Attribution 4.0 International license.

**FIG 2:**
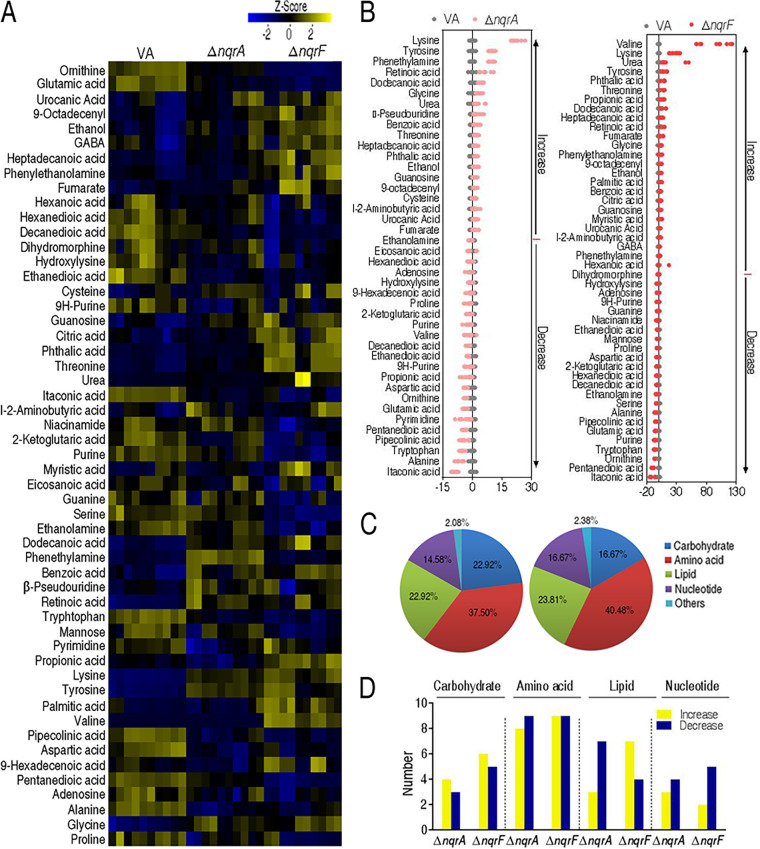
Differential abundance of metabolites in Δ*nqrA* and Δ*nqrF* strains. (A) Heat maps of a differential abundance of metabolites (row). Yellow and blue indicate an increase and decrease of the metabolites relative to the mean and standard deviation of the row metabolite level, respectively (see color scale). (B) Z-score plots of differential abundances of metabolites based on control. Data from the tested groups are separately scaled to the mean and standard deviation of the control (VA, V. alginolyticus ATCC 33787). Each point represents the value for one metabolite in one technical repeat and is colored by sample type (black, control; pink, Δ*nqrA*; red, Δ*nqrF*). (C) Category of the differential abundance of metabolites in Δ*nqrF* (left) and Δ*nqrA* (right) mutant strains. (D) Number of the differential abundance of metabolites.

Compared with the metabolome of V. alginolyticus ATCC 33787, 42 (52.5%) and 48 (60.0%) metabolites showed differential abundances (*P* < 0.05) in Δ*nqrA* and Δ*nqrF*, respectively (Fig. 2A). Z-values based on the control group were calculated, showing that it spanned from −8.82 to 22.48 in Δ*nqrA* and from −10.82 to 92.49 in Δ*nqrF* (Fig. 2B). Specifically, 23 metabolites were decreased and 19 metabolites were increased in Δ*nqrA*, and 23 metabolites were decreased and 25 metabolites were increased in Δ*nqrF*. We further examined the metabolic categories of these differential metabolites. They showed differential percentages with a ranking of amino acids > lipids > carbohydrates > nucleotides in both Δ*nqrA* and Δ*nqrF* (Fig. 2C). The numbers of up- and downregulated metabolites in these categories are shown in Fig. 2D. These results indicate that the loss of *nqrA* or *nqrF* affects bacterial metabolism.

### Enrichment of metabolic pathways involved in Δ*nqrF* and Δ*nqrA*.

**FIG 3:**
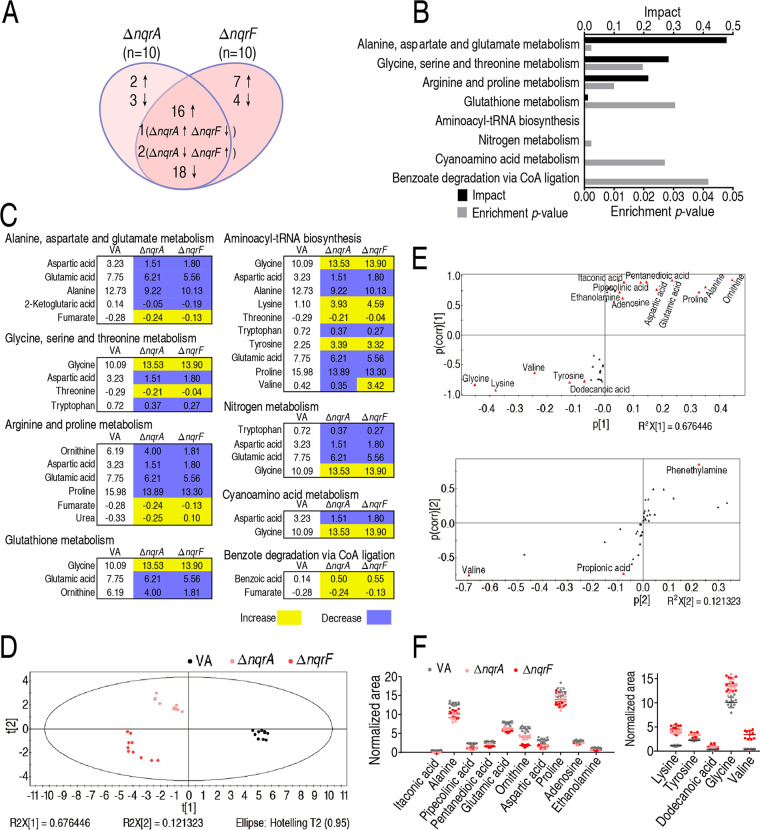
Shared pathways, metabolites, and multivariate data analysis between Δ*nqrA* and Δ*nqrF* strains. (A) Venn diagram showing the overlapping and unique differential metabolites between Δ*nqrA* and Δ*nqrF* strains. Decreased and increased metabolites are indicated with down and up arrows, respectively. (B) Enriched pathways by the 37 overlapping metabolites between the two test groups. (C) Integrative analysis of metabolites in significantly enriched pathways. Yellow and blue indicate an increase and decrease of metabolites, respectively. The number shows the relative value of differential abundance of metabolites. VA, V. alginolyticus ATCC 33787. (D) PCA analysis of control, Δ*nqrA*, and Δ*nqrF*. Each dot represents the technical replicate of samples in the plot. (E) S-plot generated from OPLS-DA. Predictive component p[1] and correlation p(corr)[1] differentiate Δ*nqrA* and Δ*nqrF* from VA. Predictive component p[2] and correlation p(corr)[2] separate Δ*nqrF* from Δ*nqrA*. The triangle represents metabolites in which candidate biomarkers are marked. (F) Scatter diagram of 15 biomarkers. *, *P* < 0.05; **, *P* < 0.01.

Furthermore, metabolic pathways of this differential abundance of metabolites were analyzed. First, a comparative analysis of the differential metabolites was performed between Δ*nqrF* and Δ*nqrA.* Among the 42 and 48 differential abundance of metabolites, respectively, in Δ*nqrA* and Δ*nqrF*, 37 metabolites overlapped, 18 were decreased, 16 were increased. Three metabolites, guanosine, propionic acid, and valine, were differentially abundant between Δ*nqrA* and Δ*nqrF*. The others were Δ*nqrA* and Δ*nqrF* specific, including 2 increased metabolites and 3 decreased metabolites in Δ*nqrA* and 7 increased metabolites and 4 decreased metabolites in Δ*nqrF* (Fig. 3A). These results indicate that most of the metabolites with differential abundance overlapped in the two mutants since they together are a part of the sodium-translocating NADH:quinone oxidoreductase.

Eight pathways were enriched by the shared differential metabolites between Δ*nqrA* and Δ*nqrF*. The most impactful pathway is alanine, aspartate, and glutamate metabolism, followed by glycine, serine, and threonine metabolism, arginine and proline metabolism, glutathione metabolism, aminoacyl-tRNA biosynthesis, nitrogen metabolism, cyanoamino acid metabolism, and benzoate degradation via coenzyme A (CoA) ligation (Fig. 3B). Except for benzoate degradation via CoA ligation, in which the two differential metabolites detected were increased in abundance, the other pathways showed most or half of the differential metabolites were decreased in abundance. Among these metabolites in these pathways, all showed the same trend of changes in abundance between Δ*nqrA* and Δ*nqrF* except for valine, which was decreased in Δ*nqrA* and increased in Δ*nqrF* (Fig. 3C). These results indicate that the loss of either *nqrA* or *nqrF* leads to similar changes in the enriched metabolic pathways.

### Identification of crucial biomarkers using multivariate data analysis.

To identify crucial biomarkers representing differential metabolomes due to the absence of *nqrA* or *nqrF*, orthogonal partial least-squares discriminant analysis (OPLS-DA) was applied for the recognition of the sample patterns, followed by ranking the altered abundance of metabolites in loading. Δ*nqrA* and Δ*nqrF* were separated from the control group clearly by predictive component 1, and the two mutants were separated by predictive component 2 (Fig. 3D). Discriminating variables were present with S-plot when cutoff values were set at greater or equal to 0.05 and 0.5 for absolute value of covariance *p* and correlation *p* (corr), respectively. Ornithine, alanine, proline, glutamic acid, aspartic acid, pentanedioic acid, itaconic acid, pipecolinic acid, ethanolamine, adenosine, dodecanoic acid, tyrosine, valine, lysine, and glycine are displayed as the metabolites that have the first 15 largest correlations and covariances in predictive component 1 between the two mutants and control. Phenethylamine, propionic acid, and valine are identified as the biomarkers that have the first three largest correlations and covariances in predictive component 2 between Δ*nqrA* and Δ*nqrF* (Fig. 3E). Among the first 15 largest correlations and covariances in predictive component 1, 5 metabolites (lysine, tyrosine, dodecanoic acid, glycine and valine) were increased and 10 metabolites (itaconic acid, alanine, pipecolinic acid, pentanedioic acid, glutamic acid, ornithine, aspartic acid, proline, adenosine, and ethanolamine) were decreased (Fig. 3F). Out of these metabolites, alanine, aspartic acid, and glutamic acid belong to alanine, aspartate, and glutamate metabolism, the biggest impact pathway. Among the three, alanine has the most absolute value of covariance *p* and the most difference in abundance between ATCC 33787 and Δ*nqrA* or Δ*nqrF.* Therefore, alanine is identified as the most crucial biomarker.

### Exogenous l-alanine promotes gentamicin-mediated killing.

**FIG 4:**
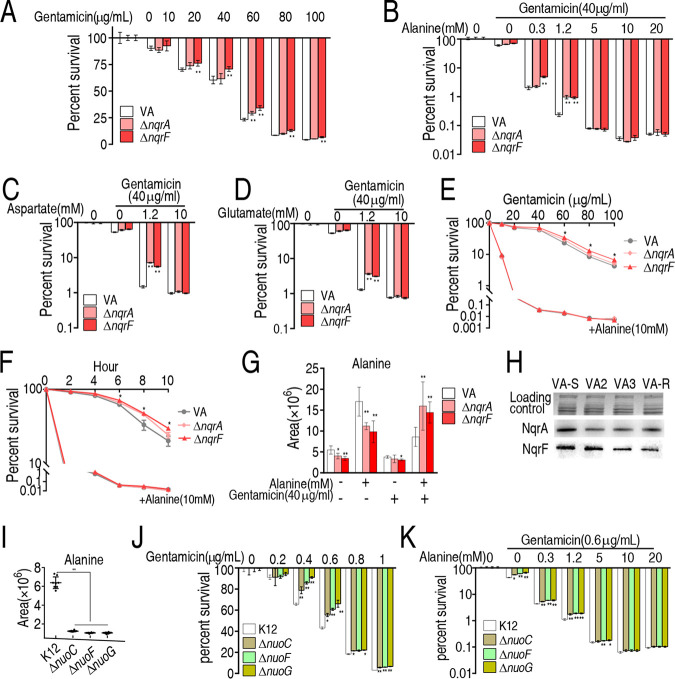
Exogenous alanine elevates sensitivity of ATCC 33787, Δ*nqrA*, and Δ*nqrF* strains to gentamicin. (A) Percent survival of V. alginolyticus ATCC 33787 (VA), Δ*nqrA*, and Δ*nqrF* in the indicated concentration of gentamicin. (B) Percent survival of VA, Δ*nqrA*, and Δ*nqrF* in the indicated concentration of alanine plus 40 μg/ml gentamicin. (C) Percent survival of VA, Δ*nqrA*, and Δ*nqrF* in the indicated concentration of aspartate plus 40 μg/ml gentamicin. (D) Percent survival of VA, Δ*nqrA*, and Δ*nqrF* in the indicated concentration of glutamate plus 40 μg/ml gentamicin. (E) Percent survival of VA, Δ*nqrA*, and Δ*nqrF* in the indicated concentration of gentamicin plus 10 mM alanine. (F) Percent survival of VA, Δ*nqrA*, and Δ*nqrF* in the presence of 40 μg/ml gentamicin and 10 mM alanine in the indicated period. (G) Abundance of alanine of VA, Δ*nqrA*, and Δ*nqrF* in the presence and/or absence of 40 μg/ml gentamicin and 10 mM alanine. (H) Western blot for NqrA and NqrF abundance of the indicated V. alginolyticus. VA2 and VA3 were clinically isolated strains. (I) Alanine abundance of Δ*nuoC*, Δ*nuoF*, Δ*nuoG*, and their parent K-12. (J) Percent survival of Δ*nuoC*, Δ*nuoF*, Δ*nuoG*, and their parent K-12 in the indicated concentration of gentamicin. (K) Percent survival of Δ*nuoC*, Δ*nuoF*, Δ*nuoG*, and their parent K-12 in the indicated concentration of alanine plus 0.6 μg/ml gentamicin. Results in panels A to G, J, and K are displayed as means ± standard errors of the means (SEM) (error bars), and significant differences are identified (*, *P* < 0.05; **, *P* < 0.01) as determined by Student’s *t* test. At least three biological repeats were carried out (panels A to G, I, J, and K).

Since the complement of depressed amino acids in the metabolome potentiates antibiotics to kill antibiotic-resistant Edwardsiella tarda, Escherichia coli, and V. alginolyticus (10, 20, 23), we supposed that exogenous alanine could cope with the resistance due to the loss of *nqrA* or *nqrF*. To demonstrate this, we first showed that higher survival was detected in Δ*nqrA* and Δ*nqrF* than in their parent strain V. alginolyticus ATCC 33787 in a gentamicin dose-dependent manner (Fig. 4A). Then, we performed the l-alanine-enabled killing of V. alginolyticus ATCC 33787, Δ*nqrA*, and Δ*nqrF*, showing higher survival of Δ*nqrA* or Δ*nqrF* than of ATCC 33787 in medium with 0.3 to 1.2 mM l-alanine, but survival returned to normal in medium with 5 to 20 mM l-alanine (Fig. 4B; Fig. S3). A similar effect but higher viability was detected in the replacement of alanine with aspartate or glutamate in the same metabolic pathway (Fig. 4C and D). Moreover, the l-alanine-enabled killing was determined in a gentamicin- and time-dependent manner. A significant difference was detected between control and Δ*nqrA* or Δ*nqrF* but not when 10 mM l-alanine was supplemented (Fig. 4E and F). The amount of alanine was quantified and compared to cells cultured in the absence of the antibiotic and alanine. Lower alanine was detected in Δ*nqrA* and Δ*nqrF* than ATCC 33787 in control, only the gentamicin group (except for Δ*nqrA*) and only alanine group, while higher alanine was measured in Δ*nqrA* and Δ*nqrF* than ATCC 33787 in the synergistic use of gentamicin and alanine group (Fig. 4G). However, 10 mM l-alanine did not change the MIC of Δ*nqrA* or Δ*nqrF* (data not shown), which may be related to the fact that MIC was measured in LB medium, which contains complex nutrients for bacterial use. We also showed that decreased abundance of NqrA and NqrF exists in clinically isolated strains VA2 and VA3 (16 and 32 MIC to gentamicin, respectively) (Fig. 4H). In E. coli, *nuoC*, *nuoF*, and *nuoG* encoding the proton-pumping NADH-ubiquinone oxidoreductase play roles similar to the roles of *nqrA* and *nqrF* NQR complex (the sodium-translocating NADH:quinone oxidoreductase) in the respiratory chain. *nuoC-*, *nuoF-*, and *nuoG-*dependent alanine depression and aminoglycoside resistance were characterized (Fig. 4I to K). Consistently, exogenous l-alanine potentiated gentamicin to kill both clinically isolated *Vibrio* species and E. coli strains (Fig. S4). These results indicate that exogenous l-alanine not only promotes the sensitivity of *Vibrio* species and E. coli to gentamicin but also reverts the gentamicin resistance due to the loss of *nqrA* or *nqrF.*

10.1128/mBio.02086-20.3 FIG S3 Percent survival of Δ*nqrA* and Δ*nqrF* and their rescued strains. Download FIG S3, PDF file, 0.2 MB. Copyright © 2020 Jiang et al. 2020 Jiang et al. This content is distributed under the terms of the Creative Commons Attribution 4.0 International license.

10.1128/mBio.02086-20.4 FIG S4 Percent survival of clinically isolated *Vibrio* species and E. coli strains in the presence of the indicated dose of gentamicin and 10 mM alanine. *Vibrio* species, ZNV10, ZNV2, ZNV4, ZNV14; E. coli, 5463, 0863, EY1, EY10, EY11; Vibrio parahaemolyticus, VP1, VP2 and VP3. Download FIG S4, PDF file, 0.2 MB. Copyright © 2020 Jiang et al. 2020 Jiang et al. This content is distributed under the terms of the Creative Commons Attribution 4.0 International license.

### l-Alanine restores levels of membrane potential, intracellular gentamicin, and activity of enzymes in the pyruvate cycle due to the loss of *nqrA* or *nqrF*.

**FIG 5:**
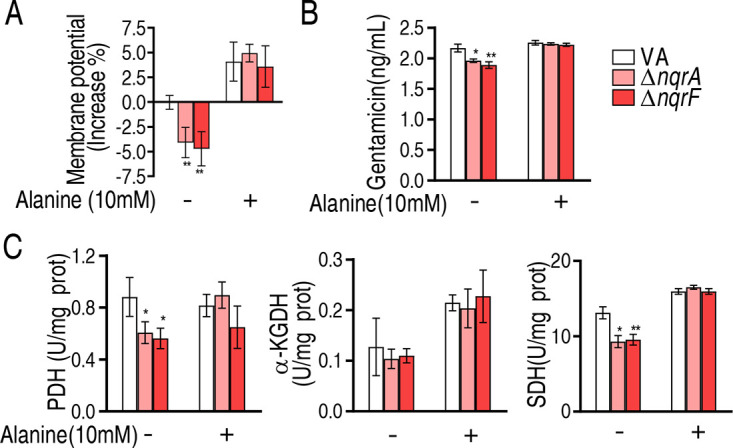
Intracellular gentamicin concentration, membrane potential, and enzyme activity in V. alginolyticus ATCC 33787 (VA), Δ*nqrA*, and Δ*nqrF* strains. (A) Membrane potential of VA, Δ*nqrA*, and Δ*nqrF* in the presence (+) or absence (−) of alanine. (B) Intracellular gentamicin of V. alginolyticus VA (ATCC 33787), Δ*nqrA*, and Δ*nqrF* in the presence or absence of alanine. (C) Activity of enzymes in the P cycle of VA, Δ*nqrA*, and Δ*nqrF* in the presence or absence of alanine. Enzyme activity is shown in units per milligram of protein. Results are displayed as means ± SEM, and significant differences are identified (*, *P* < 0.05; **, *P* < 0.01) as determined by Student’s *t* test. At least three biological repeats were carried out.

Reports have indicated that aminoglycoside antibiotic uptake is dependent on the proton motive force (PMF) (25), which motivated us to explore whether exogenous l-alanine restores the level of membrane potential due to the absence of *nqrA* or *nqrF.* To test this, the membrane potential was detected in ATCC 33787, Δ*nqrA*, and Δ*nqrF.* The loss of *nqrA* or Δ*nqrF* led to the decrease in membrane potential, but it recovered in the presence of 10 mM l-alanine (Fig. 5A). Consistently, gentamicin uptake was lower in Δ*nqrA* or Δ*nqrF* than ATCC 33787, which was reverted by the complement of the same concentration of l-alanine (Fig. 5B). We further measured the activity of pyruvate dehydrogenase (PDH), α-ketoglutarate dehydrogenase (KGDH), and succinate dehydrogenase (SDH) in the pyruvate cycle (the P cycle). The P cycle is a recently discovered cycle that provides respiratory energy, including NADH for the generation of membrane potential in bacteria (23). The activity of PDH, KGDH, and SDH was reduced in the two mutants. However, when 10 mM exogenous l-alanine was added, the activity returned to normal (Fig. 5C). These results indicate that the aminoglycoside antibiotic resistance is related to the depressed alanine abundance in Δ*nqrA* and Δ*nqrF.*

### *nqrA* and *nqrF* regulate l-alanine metabolism.

**FIG 6:**
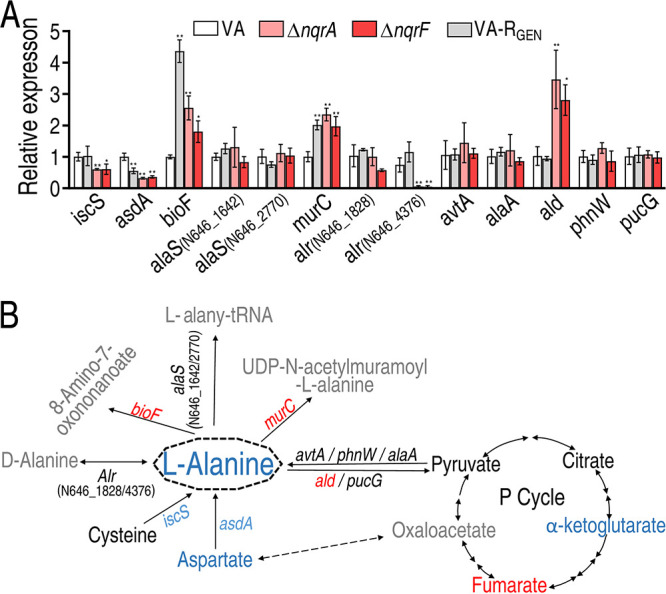
*nqrA* or *nqrF* regulates l-alanine metabolism. (A) Real-time quantitative reverse transcription-PCR (qRT-PCR) for detection of expression of genes in l-alanine metabolism. (B) Integrative analysis of metabolite abundance (data from Fig. 2A) and gene expression (data from panel A) in l-alanine metabolism of Δ*nqrA* and Δ*nqrF*. The blue and red colors denote downregulation and upregulation, respectively. Results in panel A are displayed as means ± SEM, and significant differences are identified (*, *P* < 0.05; **, *P* < 0.01) as determined by Student’s *t* test. At least three biological repeats were carried out.

We reasoned that *nqrA* and *nqrF* regulate l-alanine anabolism and/or catabolism. To test this, the expression of genes involved in l-alanine metabolism was quantified by quantitative real-time PCR (qRT-PCR). A total of 13 genes were detected. These genes degrade l-alanine to 8-amino-7-oxononanoate (*bioF*), l-alanyl-tRNA (*alaS* [N646_1642] and *alaS* [N646_2770]), and UDP-*N*-acetylmuramoyl-l-alanine (*murC*), transfer reversibly l-alanine to d-alanine (*alr* [N646_1828] and *alr* [N646_4376]) and pyruvate (*avtA*, *alaA*, *ald*, *phnW*, and *pucG*), and transform cysteine (*iscS*) and aspartate (*asdA*) to l-alanine. The increased expression of *bioF* and *murC* indicates the elevation of l-alanine catabolism, while the decreased expression of *ics* and *asdA* suggest the inhibition of l-alanine anabolism (Fig. 6). Similar gene expression was detected in lab-evolved gentamicin-resistant V. alginolyticus (VA-R_GEN_). Specifically, expression of *asdA* was decreased and expression of *bioF* and *murC* was elevated (Fig. 6A). Our recent publication showed that the P cycle is inactivated in VA-R_GEN_, consistent with the data that the P cycle does not provide aspartate for alanine biosynthesis (26). Thus, a shared l-alanine metabolism regulation exists between VA-R_GEN_ and the two mutants. These results indicate that Na^+^-NQR disruption affects l-alanine metabolism.

### Mechanisms by which *nqrA* and *nqrF* regulate l-alanine metabolism.

**FIG 7:**
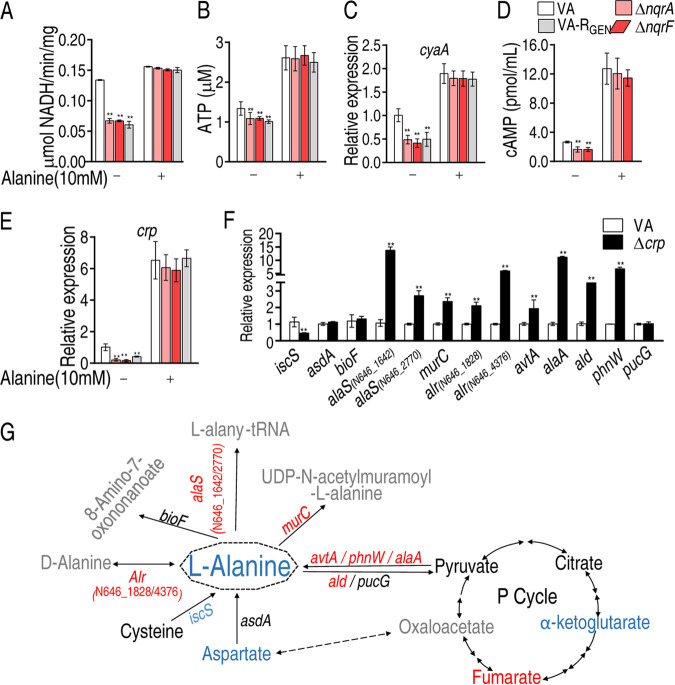
Modulation of alanine metabolism in a cAMP/CRP-dependent manner. (A) Activity of ATPase of V. alginolyticus VA (ATCC 33787), Δ*nqrA*, and Δ*nqrF* strains in the presence or absence of 10 mM alanine. (B) ATP level in V. alginolyticus VA (ATCC 33787), Δ*nqrA*, and Δ*nqrF* in the presence or absence of alanine. (C) qRT-PCR for *cyaA* expression of VA, VA-R_GEN_, Δ*nqrA*, and Δ*nqrF* in the presence or absence of alanine. (D) ELISA for the cAMP level of VA, Δ*nqrA*, and Δ*nqrF* in the presence or absence of alanine. (E) qRT-PCR for *crp* expression of VA, VA-R_GEN_, Δ*nqrA*, and Δ*nqrF* in the presence or absence of alanine. (F) qRT-PCR for gene expression of alanine metabolism in Δ*crp*. (G) Integrative analysis of metabolite abundance (data from Fig. 2A) and gene expression (data from panel F) in l-alanine metabolism of Δ*crp*. The blue and red colors denote downregulation and upregulation, respectively. Results in panels A to F are displayed as means ± SEM, and significant differences are identified (*, *P* < 0.05; **, *P* < 0.01) as determined by Student’s *t* test. At least three biological repeats were carried out.

We supposed that *nqrA* and *nqrF* regulate l-alanine metabolism through ATP since the membrane potential generated by Na^+^-NQR contributes to ATP formation via ATPase. ATPase activity and ATP content were reduced in Δ*nqrA* and Δ*nqrF* but recovered in the presence of 10 mM exogenous alanine (Fig. 7A and B). ATP is a substrate of *cyaA* for cyclic AMP (cAMP) biosynthesis. The expression of *cyaA* was reduced in Δ*nqrA* and Δ*nqrF* but returned to normal in the presence of 10 mM l-alanine (Fig. 7C). Consistently, the cAMP level was decreased and normal in the absence and presence of 10 mM l-alanine, respectively, in Δ*nqrA* and Δ*nqrF* (Fig. 7D). Similarly, the reduction of *crp* expression was detected in the two mutants, which was recovered with the addition of 10 mM alanine (Fig. 7E). Interestingly, similar results were detected in VA-R_GEN_ (Fig. 7A and C to E), suggesting a shared characteristic between VA-R_GEN_ and Δ*nqrA* or Δ*nqrF*. A further result showed that the loss of *crp* led to similar changes in gene expression as described above for Δ*nqrA* and Δ*nqrF* (Fig. 7F and G). These data support that Na^+^-NQR regulates l-alanine metabolism in a cAMP/CRP-dependent manner.

### l-Alanine restores the expression of other antiporter genes regulated by *nqrA* or *nqrF*.

**FIG 8:**
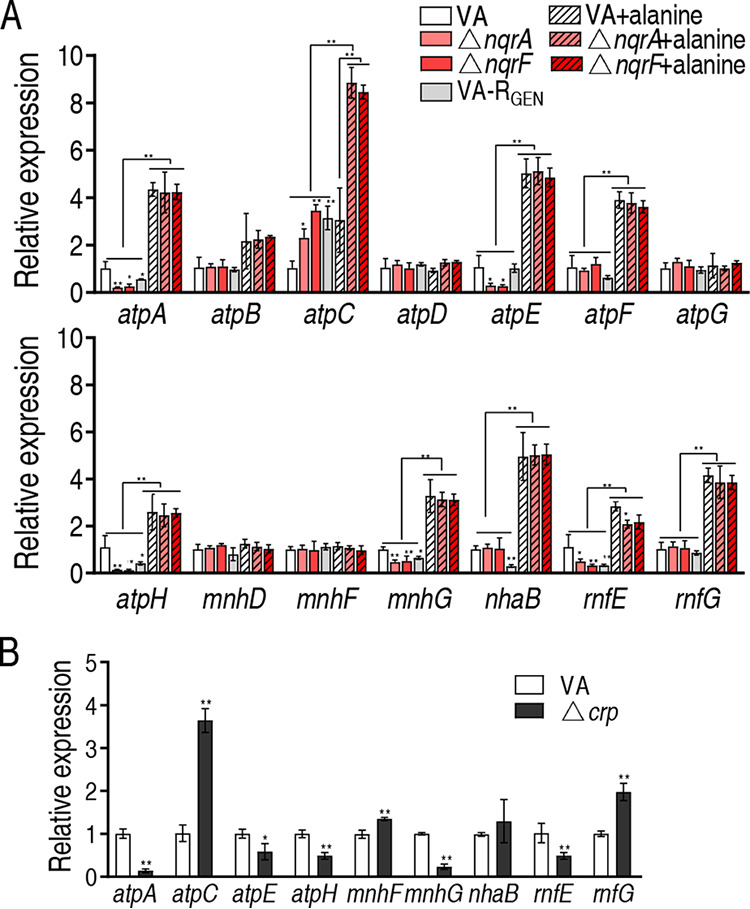
Effect of exogenous alanine on other antiporters in *nqrA-* or *nqrF*-deficient mutants and VA-R_GEN_. (A) qRT-PCR for gene expression of other antiporters in VA, VA-R_GNE_, Δ*nqrA*, and Δ*nqrF* in the presence or absence of exogenous alanine. (B) qRT-PCR for gene expression of other antiporters in VA and Δ*crp*. Results are displayed as means ± SEM, and significant differences are identified (*, *P* < 0.05; **, *P* < 0.01) as determined by Student’s *t* test. At least three biological repeats were carried out (panels A to G, I, J, and K).

**FIG 9:**
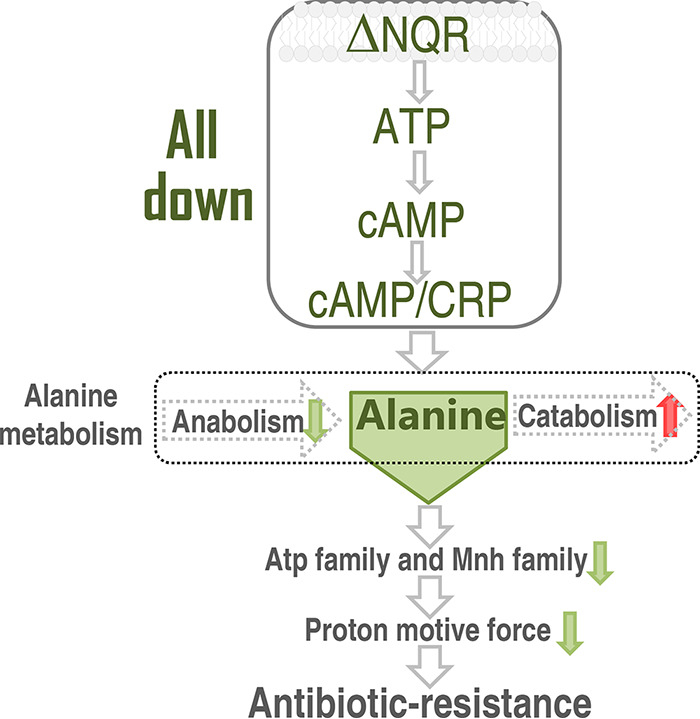
Model showing a proposed method of regulation by which Na^+^-NQR confers antibiotic resistance via the regulation of l-alanine metabolism in a cAMP/CRP-dependent manner.

We further explored how the decreased abundance of alanine modulates the membrane potential, which contributes to the intracellular gentamicin uptake. We supposed that the decrease is related to the expression of other Na^+^:H^+^ antiporters besides Na^+^-NQR. To explore this, qRT-PCR was used to detect the expression of *atpA-H* (Atp family), and *mnhD/F/G* (Mnh family), *nhaB* (Nha family), *rnfE/G* (RnfA family), of ATCC 33787, Δ*nqrA*, and Δ*nqrF* in the presence and absence of exogenous 10 mM l-alanine. Three types of differential expression were detected. (i) Lower expression was detected in Δ*nqrA* and Δ*nqrF* than in ATCC 33787, which was recovered due to the complement of 10 mM l-alanine, including *atpA*, *atpE*, *atpH*, *mnhG*, and *rntE*. (ii) Higher expression was detected in the two mutants with or without 10 mM l-alanine, but the expression was higher in the two mutants with 10 mM l-alanine than without 10 mM l-alanine, including *atpC*. (iii) Higher expression was detected in groups with 10 mM l-alanine than without 10 mM l-alanine, which is not related to whether *nqrA* or Δ*nqrF* was absent, including *nhaB* and *rnfG* (Fig. 8A). Thus, the first two types are related to the action of the increased l-alanine in Δ*nqrA* and Δ*nqrF.* Importantly, the reduced *atpA*, *atpH*, *mnhG*, and *nhaB* and the elevated *atpC* overlapped between the two mutants and VA-R_GEN_ (Fig. 8A), suggesting a shared mechanism. We further detected the expression of these differential genes of the first two types in the absence of *crp*. Consistently, the loss of *crp* reduced the expression of *atpA*, *atpE*, *atpH*, *mnhG*, *rnfE* and elevated the expression of *atpC* (Fig. 8B). These results indicate that the regulation of *nqrA* or *nqrF* to other Na^+^:H^+^ antiporters, which is under the control of cAMP-CRP, is related to l-alanine abundance and gentamicin resistance. A model showing a proposed way that Na^+^-NQR confers antibiotic resistance via the regulation of l-alanine metabolism in a cAMP/CRP-dependent manner is shown in Fig. 9.

## DISCUSSION

In the present study, we found that the absence of *nqrA* or *nqrF* leads to elevated MIC to aminoglycoside antibiotics, including amikacin, gentamicin, and kanamycin in V. alginolyticus. Na^+^-NQR functions as a unique redox-driven sodium pump, playing a vital role in the electron transport chain and sodium motive force (14). The sodium motive force is similar to the proton motive force (PMF), which is generated by a redox-driven proton pump out of Na^+^ (25, 27). It is documented that PMF contributes to aminoglycoside antibiotic uptake (25). Therefore, the elevated MIC of *nqrA*- and *nqrF*-deleted mutants to aminoglycoside antibiotics is attributed to the decrease of PMF. However, whether Na^+^-NQR exhibits action through other indirect ways to regulate the generation of PMF and thereby confer antibiotic resistance is still unknown.

Na^+^-NQR plays a key role in energy metabolism as a unique redox-driven sodium pump (14). Recent metabolism analysis has shown the increased reductive pathway of the tricarboxylic acid (TCA) cycle and decreased purine metabolism in V. cholerae Δ*nqrA-F* mutant (17), which suggests that Na^+^-NQR regulates other metabolisms besides the action of the Na^+^-NQR pump. However, whether Na^+^-NQR confers antibiotic resistance through the other metabolisms is still unknown. A line of evidence has demonstrated that the bacterial metabolic environment confounds antibiotic susceptibility and resistance (10, 23, 28). Therefore, the present study used GC-MS-based metabolomics to explore differential metabolomes due to the loss of *nqrA* or *nqrF* and to identify key metabolic pathways and crucial metabolites as biomarkers for further understanding of the role of Na^+^-NQR in antibiotic resistance. Our results showed that the most impacted pathway is alanine, aspartate, and glutamate metabolism. Among the detected metabolites in the pathway, aspartic acid, glutamic acid, alanine, and 2-oxoglutaric acid were reduced, where the first three were identified as crucial biomarkers. Out of the three, alanine had the most absolute value of covariance *p*. Besides, alanine also exhibits the most difference in abundance between ATCC 33787 and Δ*nqrA* or Δ*nqrF.* Thus, alanine was selected as the most impacted metabolite. These results are consistent with our recent data that the most strongly impacted KEGG pathway is alanine, aspartate, and glutamate metabolism and the decreased abundance of alanine and glutamate was observed, with the high impact on alanine in kanamycin-resistant Edwardsiella tarda (10, 23). These findings suggest that Na^+^-NQR regulates alanine, aspartate, and glutamate metabolism to cope with aminoglycoside antibiotics, where alanine is especially crucial. The conclusion is supported by the replacement of alanine with aspartate or glutamate, which showed a similar trend but lower efficacy. In addition, the alanine-mediated regulation was characterized in E. coli *nuoC*, *nuoF*, and *nuoG*, which play a role similar to the role of the NQR complex. Therefore, aminoglycoside potentiation of alanine may be conservative in bacteria.

Compared with their parent strain ATCC 33787, Δ*nqrA* and Δ*nqrF* showed significant resistance to antibiotics. Our recent reports have indicated that antibiotic-resistant bacteria have an antibiotic-resistant metabolome, which can be reverted by crucial biomarkers into an antibiotic-susceptible metabolome and thereby the bacteria become sensitive to the antibiotics (10, 23). The reversion is termed a reprogramming metabolome (28). To explore the possibility that *nqrA* and *nqrF* confer the antibiotic resistance via a l-alanine-related metabolome, exogenous l-alanine was used to reprogram the resistant metabolome of Δ*nqrA* and Δ*nqrF* to a susceptible metabolome. Exogenous l-alanine potentiated antibiotics to kill Δ*nqrA* and Δ*nqrF* by approximately 1,700- and 1,800-fold, respectively. Higher survival was detected in Δ*nqrA* and Δ*nqrF* than V. alginolyticus ATCC 33787 in medium with 0.3 to 2.5 mM l-alanine but not in medium with 5 to 20 mM l-alanine, suggesting that the high abundance of l-alanine reverts the resistance resulting from the loss of *nqrA* and *nqrF*. GC-MS analysis also demonstrated that the synergistic use of alanine and gentamicin promoted intracellular concentration of alanine in Δ*nqrA* and Δ*nqrF*. These results support the conclusion that *nqrA* and *nqrF* regulate l-alanine metabolism to play a role in antibiotic resistance.

The present study further revealed that both *nqrA* and *nqrF* positively and negatively regulate gene expression of l-alanine anabolism and catabolism, respectively. Regulation proceeds by the events that the loss of *nqrA* or *nqrF* decreases ATPase activity and ATP and cAMP levels and thereby the cAMP/CRP complex is inhibited. The inhibition reduces and promotes the transformation and degradation of l-alanine, respectively. Similar results are obtained in Δ*crp*. Importantly, the changes in alanine metabolism and the cAMP/CRP complex are overlapped between VA-R_GEN_ and Δ*nqrA* and Δ*nqrF*. The findings characterize a previously unknown Na^+^-NQR-dependent antibiotic resistance mechanism that the reduced Na^+^-NQR confers antibiotic resistance via reducing l-alanine in a cAMP/CRP-dependent manner, highlighting a way to further understand the role of Na^+^-NQR in antibiotic resistance. As noted in Results, some data are different between Δ*nqrA* and Δ*nqrF*, such as MIC to amikacin and three reversals of the abundance of metabolites. These results suggest that the two genes are linked to their respective roles except for the shared function.

At last, the present study further explores why the reduced l-alanine confers the antibiotic resistance due to the absence of *nqrA* or *nqrF.* Exogenous l-alanine recovers the decreased membrane potential and intracellular gentamicin concentration resulted from the loss of Δ*nqrA* or Δ*nqrF*, suggesting that l-alanine promotes the membrane potential via other Na^+^:H^+^ antiporters rather than Na^+^-NQR. In V. alginolyticus cells, five Na^+^:H^+^ antiporters are included. They are NQR family, Nha family, Mnh family, Rnf family, and Atp family (29–34). The expression of Atp family *atpA*, *atpE*, *aptH* and Mnh family *mnhG* were lower when the NQR family did not work, most of which are overlapped in VA-R_GNE_ and are under the control of cAMP/CRP. However, exogenous 10 mM l-alanine recovered the decrease. Similarly, expression of *atpC* was higher in the two mutants than control with or without 10 mM l-alanine, but expression was higher in the two mutants with exogenous 10 mM l-alanine than without exogenous 10 mM l-alanine. Minato et al. found that lack of Na^+^-NQR did not affect any of the Na^+^-pumping-related phenotypes of V. cholerae in studying metabolism, motility, and osmotic stress resistance and speculated that other secondary Na+ pump(s) can compensate for the Na^+^-pumping activity of Na^+^-NQR (35). These results indicate that *nqrA* and *nqrF* regulate Atp family and Mnh family via l-alanine abundance in V. alginolyticus. Also, the *atpA* and *aptC* genes are regulated by a single promoter as an operon (36), but the differential expression is detected in Δ*nqrA* or Δ*nqrF*. Possibly, this is related to regulation at the transcript level.

In summary, the present study showed the loss of *nqrA* or *nqrF* leads to the elevated resistance to aminoglycoside antibiotics. The losses caused similar differential metabolomes, characterizing alanine, aspartate, and glutamate metabolism and decreased alanine as the most impacted pathway and the crucial biomarker, respectively. These results indicate that the metabolic changes, especially l-alanine metabolism, are related to the aminoglycoside resistance. *nqrA* and *nqrF* regulate l-alanine metabolism in a cAMP/CRP-dependent manner. The regulation affects Atp family and Mnh family to generate membrane potential and promote gentamicin uptake. These findings extend our understanding of the action of Na^+^-NQR in antibiotic resistance.

## MATERIALS AND METHODS

### Bacterial strain and culture conditions.

Bacterial strains used in this study were from the collection of our laboratory and listed in Table S1 in the supplemental material. The antimicrobial agents amikacin, gentamicin, and kanamycin were purchased from a commercial source (Shanghai Sangon Biological Engineering Technology & Services Co. Ltd., China). A single colony was propagated in 3% NaCl Luria-Bertani (LB) (tryptone [10 g/liter], yeast extract [5 g/liter], NaCl [30 g/liter]) broth for 8 h at 30°C. The cultures were diluted to 1:100 using fresh 3% NaCl LB medium and grown at 30°C.

10.1128/mBio.02086-20.5 TABLE S1 Bacterial strains used in this study. Download Table S1, DOCX file, 0.01 MB. Copyright © 2020 Jiang et al. 2020 Jiang et al. This content is distributed under the terms of the Creative Commons Attribution 4.0 International license.

### Construction and complementation for genetically modified mutants with *nqrA* or *nqrF* deleted.

Primers were designed according to Table S2 using CE Design V1.03 software. The upstream and downstream 500-bp fragments were first amplified from the genome using two pairs of primers, primers P1 and P2 and primers P3 and P4 and then merged into a 1,000-bp fragment by overlap PCR using a pair of primers, primers P1 and P4. After the fragments were digested by SacI and XbaI, they were ligated into the pRE112 vector digested by the same enzymes and transformed into MC1061 competent cells. The plasmids were identified by PCR using a pair of primers, primers P1 and P4, and sequenced. The sequenced plasmids were transformed into S17 competent cells. S17 and recipient bacterium V. alginolyticus ATCC 33787 were cultured to an optical density (OD) of 1.0 and then mixed at a ratio of 4:1. After centrifugation, the mixtures were resuspended with LB medium, dropped onto sterilized filter paper soaked with LB medium, and cultured for 16 h at 30°C. All the bacteria rinsed from the filter paper with LB medium were smeared onto the LB plates with ampicillin (100 μg/ml) and chloramphenicol (30 μg/ml). After the bacteria were identified by plasmid PCR using a pair of primers P1 and P4 and sequencing, the bacteria were cultured and smeared onto the LB plates with 20% sucrose. The clones were cultured and smeared onto the LB plates with 20% sucrose or chloramphenicol. The clones, which did not grow on the LB plates with chloramphenicol but grew on the LB plates with 20% sucrose, were identified by PCR using primer P7P8, P4P7, or P5P6 for further use. For gene complementation, the entire coding regions of *nqrA* and *nqrF* were amplified by PCR and cloned into the PACYC184 plasmid. Primers are listed in Table S3. The recombinant plasmids were transformed into the Δ*nqrA* and Δ*nqrF* mutant strains and selected on Luria broth with 25 μg/ml chloramphenicol to construct the complemented mutant strains +*nqrA* and +*nqrF*.

10.1128/mBio.02086-20.6 TABLE S2 Primers for genetically modified mutants with *nqrA* or *nqrF* deleted. Download Table S2, DOC file, 0.04 MB. Copyright © 2020 Jiang et al. 2020 Jiang et al. This content is distributed under the terms of the Creative Commons Attribution 4.0 International license.

10.1128/mBio.02086-20.7 TABLE S3 Primers for gene complementation. Download Table S3, DOC file, 0.04 MB. Copyright © 2020 Jiang et al. 2020 Jiang et al. This content is distributed under the terms of the Creative Commons Attribution 4.0 International license.

### Measurement of MIC.

MIC was determined by antimicrobial susceptibility testing according to CLSI guidelines. In brief, the overnight bacteria cultured in 3% NaCl LB medium were diluted 1:100 (vol/vol) in fresh 0.5% yeast broth and cultured at 30°C to an optical density at 600 nm (OD_600_) of 0.5. Then 10 μl of 0.5 × 10^5^ CFU wsd added into each well of a 96-well microtiter polystyrene tray with 100 μl of a series of twofold dilutions of an antibiotic. The mixtures were incubated at 30°C for 16 h. MIC was defined as the lowest antibiotic concentration that inhibited visible growth. Three biological repeats were carried out.

### Measurement of the growth curve.

V. alginolyticus ATCC 33787 and its mutants Δ*nqrA* and Δ*nqrF* were separately cultured in 3% NaCl LB medium overnight and were diluted 1:100 (vol/vol) in fresh 3% NaCl LB broth. These bacteria were cultured at 30°C with shaking at 200 rpm and monitored at 0.5, 1, 1.5, 2, 4, 6, 8, 10, 12, 14, and 24 h through measurement of OD_600_. All experiments were carried out in biological triplicates.

### Metabolomic analysis.

Sample preparation was performed as described previously (26). In brief, bacterial cells were collected in the exponential phase (OD_600_ of 0.5) by centrifugation at 8,000 rpm for 5 min at 4°C. Cellular metabolites were extracted with 500 μl of cold methanol, which contained 10 μl of 0.2 mg/ml ribitol (Sigma) as an analytical internal standard. Cells were lysed by sonication for 3 min at 30% intensity and were centrifuged for 10 min at 12,000 rpm at 4°C. Then, 500 μl of supernatant was transferred into a new 1.5-ml tube and dried in a rotary vacuum centrifuge device (Labconco). The resulting samples were used for GC-MS analysis. Each sample had five biological replicates with two technical repeats.

GC-MS analysis was carried out with a variation on the two-stage technique as previously described (37). First, protected carbonyl moieties of samples were exposed through a 90-min 37°C reaction with 80 μl of 20 mg/ml methoxyamine hydrochloride in pyridine. This was followed by the derivatization of acid protons by a 30-min 37°C reaction with the addition of 80 μl of *N*-methyl-*N*-trimethylsilytrifluoroacetamide (MSTFA) (Sigma). Chemical analysis of samples was carried out by an Agilent G1701EA GC-MSD ChemStation (Agilent). The injection port was maintained at 270°C. The derivatized sample of 1 μl was injected into a dodecyl benzene sulfonic acid (DBS) column (30-m length, 250-μm inner diameter [i.d.], 0.25-μm thickness) using the splitless mode. The MS source temperature was maintained at 250°C in the electron ionization (EI) (ionized directly) mode at 70 eV ionization energy and with 8,000 V acceleration voltage. The MS quad temperature was held constant at 150°C. The initial temperature of the GC oven was programmed at 85°C for 3 min, followed by an increase to 285°C at a rate of 5°C min^−1^. Then, the temperature was increased to 310°C at a rate of 20°C min^−1^ and held for 7 min. Helium was used as the carrier gas. The flow was kept constant at 1 ml min^−1^. The MS was operated in a range of 50 to 600 *m/z*.

Multivariate statistical analyses were applied for metabolites. For each compound, a Mann-Whitney U-test was performed to detect significant differences between control and test groups. The compounds that matched by retention time and mass spectrum were merged in each sample. Correlation between the compounds was determined using the Spearman correlation coefficient. Normalization and analysis of metabolomic data were completed using Microsoft Excel. The metabolite abundance data matrix was normalized by the quantity of added internal standards and the total intensity. Z-score and hierarchical clustering were used to analyze the normalization area. Normalized data were used for hierarchical clustering in the R platform with the package “gplots,” using the distance matrix calculated by the Euclidean method. Principal-component analysis (PCA) was used to reduce the high dimension of the data set and analyze the covariance variation and emphasize the outlier in clustering. Pathway analysis was performed by MetPA, which contains pathway enrichment analysis and pathway topological analysis. Pathway enrichment analysis determines which metabolic pathways have compounds that are overrepresented and have significant perturbations to their concentrations, while MetPA employs some topological assessment tools to measure centrality or “hubness” objectively, termed pathway impact. Pathway impact is a combination of the centrality and pathway enrichment results (https://en.wikipedia.org/wiki/Metabolomic_Pathway_Analysis).

### Antibiotic bactericidal assay.

The antibiotic bactericidal assay was performed as previously described (10, 38). In brief, a single colony was propagated in 50 ml of LB broth in a 250-ml flask for 14 h at 30°C in a shaker. The cultures were collected by centrifugation at 8,000 rpm for 5 min. The samples were washed three times with 30 ml sterile saline, resuspended in M9 minimal medium (M9) supplemented with 10 mM acetate, 2 mM MgSO_4_, and 100 μM CaCl_2_, and then diluted to an OD_600_ of 0.2. Metabolite and/or antibiotics were added and incubated at 30°C and 200 rpm for 6 h. To determine bacterial counts at specified time points, 100-μl portios of samples were removed and serially diluted. An aliquot of 10 μl of each dilution was spot plated onto LB agar plates and cultured at 30°C for 12 h to determine CFU. Only dilutions that yielded 20 to 200 colonies were available. Percent survival was determined by dividing the CFU obtained from a treated sample by the CFU obtained from a control sample.

### Measurement of membrane potential.

Measurement of membrane potential was performed as previously described (39). In brief, a BacLight bacterial membrane potential kit (Invitrogen) was used to assess changes in membrane potential of V. alginolyticus ATCC 33787, Δ*nqrA*, and Δ*nqrF* incubated with or without 10 mM alanine. 10^6^ CFU of bacteria were used and stained with 10 μl of 3 mM 3,3'-diethyloxacarbocyanine iodide [DIOC_2_(3)], followed by incubation for 30 min. Samples were analyzed using a FACSCalibur flow cytometer (Becton Dickinson, San Jose, CA) using the following settings: fluorescein isothiocyanate (FITC) voltage, 250 V; mCherry voltage, 650 V; forward scatter (FSC) threshold, 1,000; recorded events, 100,000. The red/green (mCherry/FITC) values for each cell were determined, normalized, and then compared between samples. The relative PMF of test samples was determined.

### Measurement of activity of enzymes in the pyruvate cycle.

The measurement of enzyme activity was carried out as previously described (22). The cells were incubated in the presence of gentamicin with 10 mM alanine or without 10 mM alanine at 30°C for 6 h, collected, and washed three times. The bacterial cells were suspended in 250 μl of phosphate-buffered saline (PBS) and disrupted by sonic oscillation. After centrifugation, supernatants were collected. The protein concentration of the supernatant was determined using the BCA protein assay kit (Beyotime Inc., China). The activity of succinate dehydrogenase (SDH), pyruvate dehydrogenase (PDH) and α-ketoglutarate dehydrogenase (KGDH) was detected as follows. Supernatant (120 μl) containing 0.2 mg protein was transferred to a SDH reaction mix [0.5 mM 3-(4,5-dimethylthiazol-2-yl)-2,5-diphenyltetrazolium bromide (MTT), 2.5 mM MgCl_2_, 6.5 mM phenazine methosulfate (PMS), 5 mM sodium succinate, 50 mM PBS] or PDH/KGDH reaction mix (0.5 mM MTT, 2.5 mM MgCl_2_, 6.5 mM PMS, 0.2 mM thiamine pyrophosphate [TPP], 2 mM sodium pyruvate/5 mM alpha-ketoglutaric acid potassium salt, 50 mM PBS) to a final volume of 200 μl in a 96-well plate. After incubation at 30°C for minutes, the absorbance at 566 nm was recorded. Enzyme activity was calculated according to a standard curve.

### Measurement of ATPase activity.

To quantify intracellular F-type ATPase activity, a single colony was propagated in 50 ml LB broth in 250-ml flasks for 14 h at 30°C. Bacterial cells were collected and suspended to an OD_600_ of 0.6 in M9 medium and incubated with or without 10 mM l-alanine at 30°C for 6 h. A 10-ml sample with OD_600_ of 1.0 was dissolved in 1 ml PBS buffer (pH 7.4) and disrupted by sonication (20% output, 2-s ultrasound, and 3-s interval time for a total of 3 min) in an ice bath. Total proteins of 100 μg were added for the measurement of F-type ATPase activity with F-type ATPase activity assay kit (catalog no. GMS50248.2; Genmed, Shanghai, China). The intracellular enzyme activity unit was quantified according to the manufacturer’s manual.

### Measurement of ATP.

BacTiter-Glo microbial cell viability assay (Promega Corporation, Madison, WI, USA) was used to quantify the intracellular concentration of ATP. Cells were suspended to an OD_600_ of 0.6 in M9 medium and incubated with or without 10 mM l-alanine at 30°C for 6 h. The cell extract (50 μl) was used to determine ATP measurement by using luciferin/luciferase and BacTiter-Glo microbial cell viability assay.

### Detection of intracellular gentamicin concentration.

Intracellular gentamicin was detected as previously described (39). Gentamicin enzyme-linked immunosorbent assay (ELISA) rapid diagnostic kit (Beijing Clover Technology Group Inc., Beijing, China) was used to assess intracellular gentamicin concentration. Cells were suspended to an OD_600_ of 0.6 in M9 medium and incubated in the presence of gentamicin with or without 10 mM alanine at 30°C for 6 h. The cells were collected and washed three times with sterile saline. The resulting cells were suspended with M9 medium and adjusted to an OD_600_ of 1.0. An aliquot of 10 ml was sonicated for 3 min. The resulting supernatant was collected for the detection of gentamicin following the instructions of the gentamicin ELISA rapid diagnostic kit.

### qRT-PCR.

Quantitative real-time PCR (qRT-PCR) was carried out as described previously (40). Total RNA was isolated from V. alginolyticus using TRIzol reagent (Invitrogen Life Technologies) according to the protocol. Electrophoresis in 1% (wt/vol) agarose gels was performed to check the quality of extracted RNA. By using a PrimeScript RT reagent kit with gDNA eraser (TaKaRa, Japan), reverse transcription-PCR was carried out on 1 μg of total RNA according to the manufacturer’s instructions. Primers are listed in Table S4. qRT-PCR was performed in 384-well plates with a total volume of 10 μl, and the reaction mixtures were run on a LightCycler 480 system (Roche, Germany). Data are shown as the relative mRNA expression compared with control with the endogenous reference 16S rRNA gene.

10.1128/mBio.02086-20.8 TABLE S4 Primers for qRT-PCR. Download Table S4, DOC file, 0.1 MB. Copyright © 2020 Jiang et al. 2020 Jiang et al. This content is distributed under the terms of the Creative Commons Attribution 4.0 International license.

### Measurement of cAMP.

cAMP Complete ELISA kit (Enzo Biochem Inc., New York, NY, USA) was used to assess the concentration of cAMP within bacterial cells. Cells were incubated in medium with or without alanine, collected, and washed three times. The resulting cells were collected for detection of cAMP following the instructions of the cAMP Complete ELISA kit.
