# Supplementary figures and images for: Na+-NQR Confers Aminoglycoside Resistance via the Regulation of l-Alanine Metabolism
Source: mBio. 2020 Nov 17;11(6):e02086-20. doi: 10.1128/mBio.02086-20 (PMC7683393; doi:10.1128/mBio.02086-20)

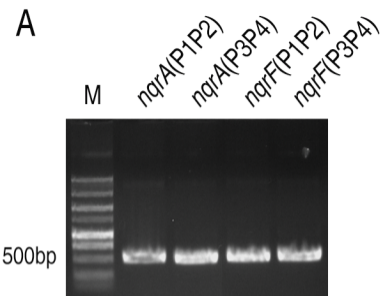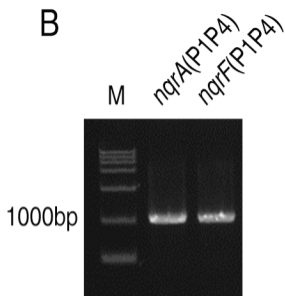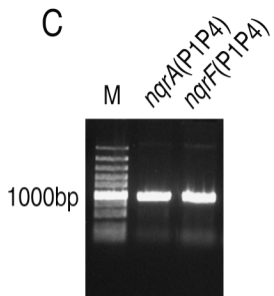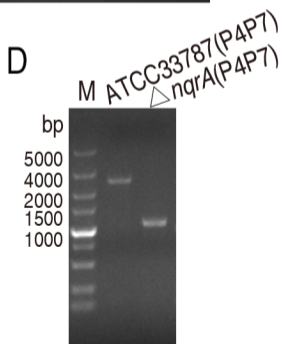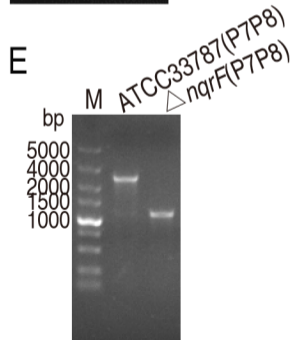

Supplement: FIG S1 [file mBio.02086-20-sf001.pdf]

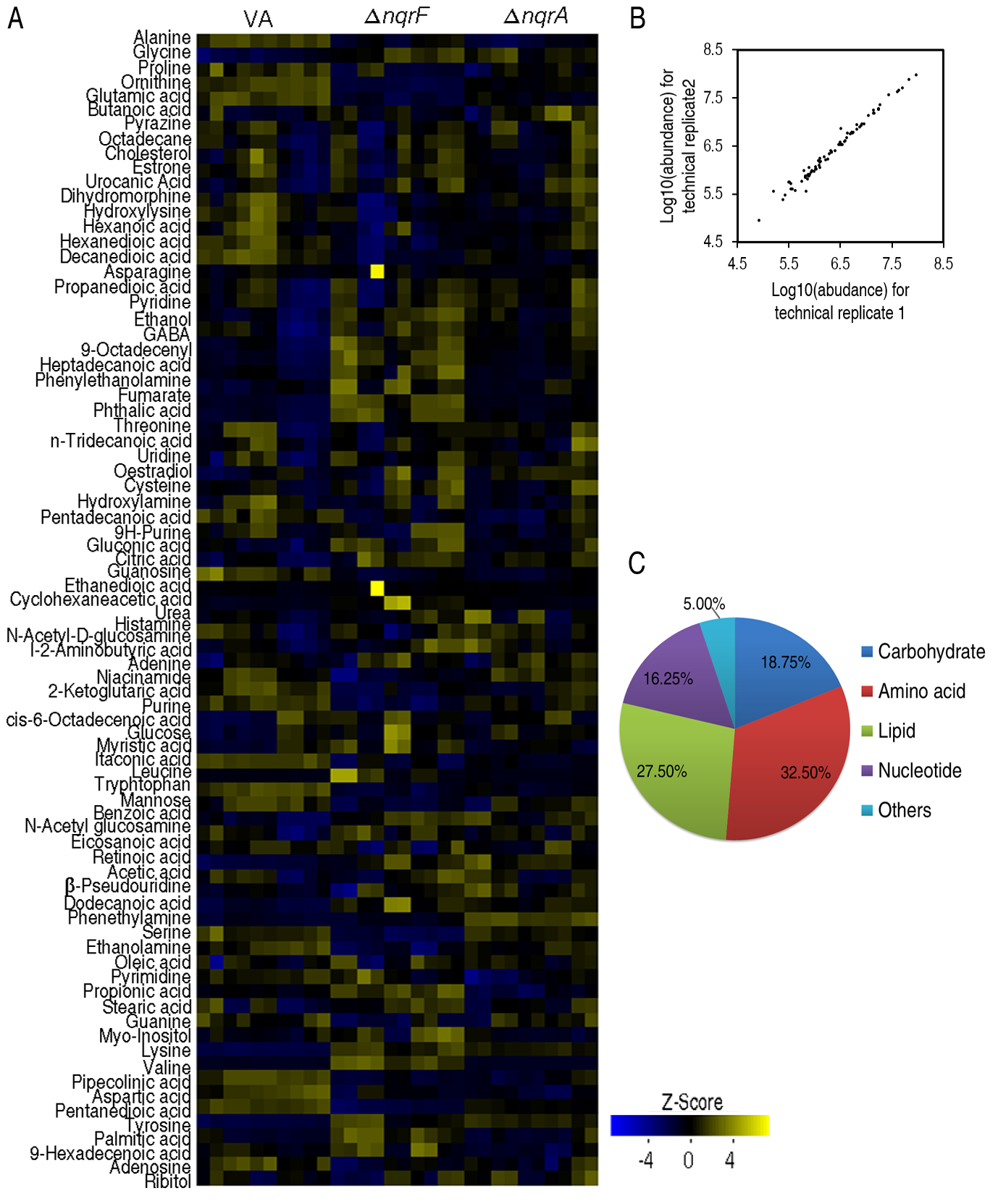

Supplement: FIG S2 [file mBio.02086-20-sf002.tif]

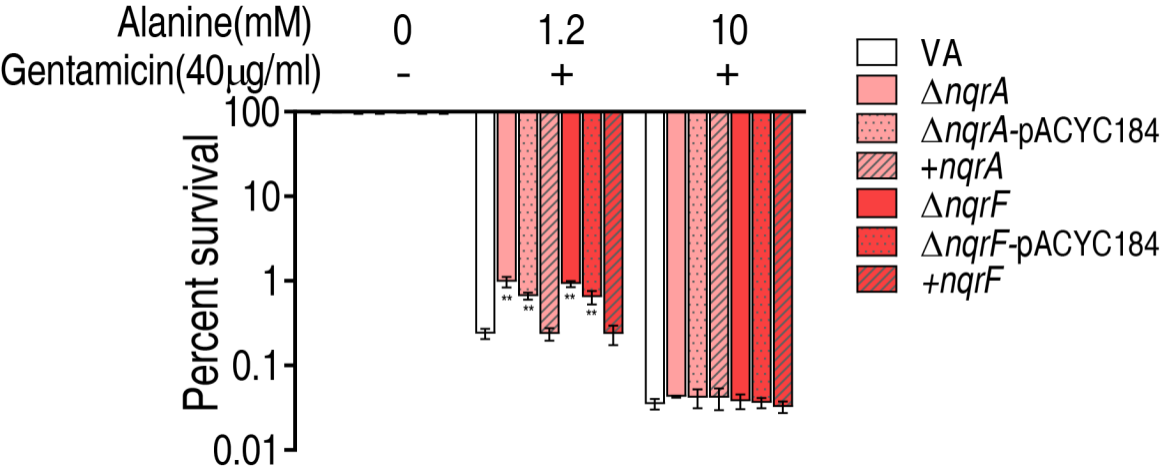

Supplement: FIG S3 [file mBio.02086-20-sf003.pdf]

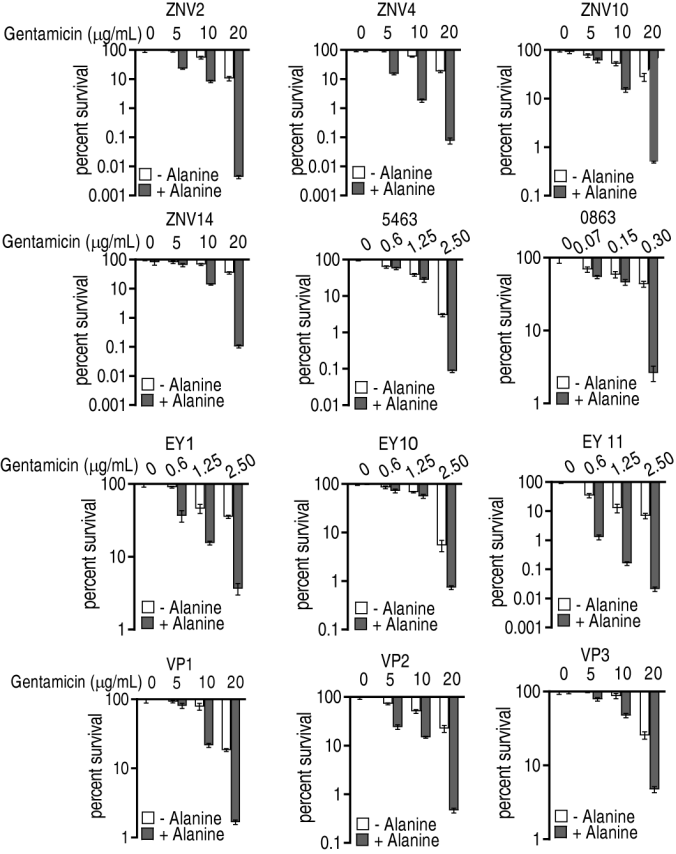

Supplement: FIG S4 [file mBio.02086-20-sf004.pdf]
