# Supplementary material for: Na+-NQR Confers Aminoglycoside Resistance via the Regulation of l-Alanine Metabolism
Source: mBio. 2020 Nov 17;11(6):e02086-20. doi: 10.1128/mBio.02086-20 (PMC7683393; doi:10.1128/mBio.02086-20)
Supplement: TABLE S1 [file mBio.02086-20-st001.docx]

**Supplementary Tab. 1 Bacterial strains used in the study**

| Strain | | Original |
| --- | --- | --- |
| *E. coli* | *E. coli* K12 | KEIO collection |
|  | Δ*nuoC* | KEIO collection |
|  | Δ*nuoF* | KEIO collection |
|  | Δ*nuoG* | KEIO collection |
|  | 5463 | The collections of our laboratory, isolated from patient |
|  | 863 | The collections of our laboratory, isolated from patient |
|  | EY1 | The collections of our laboratory, isolated from patient |
|  | EY10 | The collections of our laboratory, isolated from patient |
|  | EY11 | The collections of our laboratory, isolated from patient |
| *Vibrio species* | *V. alginolyticus* ATCC33787 | The collections of our laboratory |
|  | VA-RGEN | The collections of our laboratory |
|  | Δ*nqrA* | This study |
|  | Δ*nqrF* | This study |
|  | Δ*nqrA-*pACYC184 | This study |
|  | Δ*nqrF-*pACYC184 | This study |
|  | +*nqrA* | This study |
|  | *+nqrF* | This study |
|  | Δ*crp* | The collections of our laboratory |
|  | *V.parahaemolyticus,* VP1 | The collections of our laboratory, isolated from fish |
|  | *V.parahaemolyticus,* VP2 | The collections of our laboratory, isolated from fish |
|  | *V.parahaemolyticus,* VP3 | The collections of our laboratory, isolated from fish |
|  | ZNV2 | The collections of our laboratory, isolated from seafood |
|  | ZNV4 | The collections of our laboratory, isolated from seafood |
|  | ZNV10 | The collections of our laboratory, isolated from seafood |
|  | ZNV14 | The collections of our laboratory, isolated from seafood |
