# Supplementary material for: Na+-NQR Confers Aminoglycoside Resistance via the Regulation of l-Alanine Metabolism
Source: mBio. 2020 Nov 17;11(6):e02086-20. doi: 10.1128/mBio.02086-20 (PMC7683393; doi:10.1128/mBio.02086-20)
Supplement: TABLE S2 [file mBio.02086-20-st002.doc]

**Supplementary Tab. 2** Primers for genetically modified mutants of *nqrA* or *nqrF* deletion

| **Gene** | **Primer sequence (5'-3')** |
| --- | --- |
| *nqrF*-1F | catgaattcccgggagagctcCAGTTGTGGTAGTTCTGACTATCGC |
| *nqrF*-2R | ggaggaattaTGACTATTCCTTAATTGTTGCGGT |
| *nqrF*-3F | ggaatagtcaTAATTCCTCCAAGTGATTGAATCTATG |
| *nqrF*-4R | cgatcccaagcttcttctagaTCACATCCAATGCACCTAAAGTAAG |
| *nqrF*-5F | TGGCGATCGTTACACAACCA |
| *nqrF*-6R | TCACGCGCTTCACCTTTAGT |
| *nqrF*-7F | GGAACATTACATTAGTCTGTTAGTTAAA |
| *nqrF*-8R | ACCATCAACAGATAGGTGGTGAATA |
| *nqrA*-1 F | catgaattcccgggagagctcCCGATGTGTATGGGTGGCG |
| *nqrA*-2R | gaaaattaACGCACTTGCACTACTTTTATCGG |
| *nqrA*-3F | gtgcaagtgcgtTAATTTTCATGGCTCTTAAAAAGTTTC |
| *nqrA*-4R | cgatcccaagcttcttctagaGATAGAAGTTACAAAGAAACCTTCGTTAA |
| *nqrA*-5F | CTGGGCAATGCCTGGTAAGA |
| *nqrA*-6R | ACGAAGCAGCAAAGTAGGCT |
| *nqrA*-7F | AATTTTGGCAGATGAACTGGATAAC |
| *nqrA*-8R | CTGTACCACCGAAGATCTCTTTTGC |
