# Supplementary material for: Na+-NQR Confers Aminoglycoside Resistance via the Regulation of l-Alanine Metabolism
Source: mBio. 2020 Nov 17;11(6):e02086-20. doi: 10.1128/mBio.02086-20 (PMC7683393; doi:10.1128/mBio.02086-20)
Supplement: TABLE S3 [file mBio.02086-20-st003.doc]

**Supplementary Tab.3** Primers for gene complementation

| **Gene** | **Primer** | **Primer sequence (5'-3')** |
| --- | --- | --- |
| *nqrA* | Forward | aacgcagtcaggcaccgtgtatgattacaataaagaagggct |
|  | Reverse | gaggtgccgccggcttccatttacccttccttctcgatag |
| *nqrF* | Forward | aacgcagtcaggcaccgtgtatggacattattcttggtgt |
|  | Reverse | gaggtgccgccggcttccatttaaccaccgaagtcatcta |
