# Supplementary material for: Na+-NQR Confers Aminoglycoside Resistance via the Regulation of l-Alanine Metabolism
Source: mBio. 2020 Nov 17;11(6):e02086-20. doi: 10.1128/mBio.02086-20 (PMC7683393; doi:10.1128/mBio.02086-20)
Supplement: TABLE S4 [file mBio.02086-20-st004.doc]

**Supplementary Tab. 4** Primers for QRT-PCR

| **Gene** | **KEGG entry** | **Primer** | **Primer sequence (5'-3')** |
| --- | --- | --- | --- |
| 16S rRNA |  | Forward | GGGAGTACGGTCGCAAGAT |
|  |  | Reverse | GCTGGCAAACAAGGATAAGG |
| *iscS* | N646_2754 | Forward | TTAGAAGCAGTGACGGTAAACGG |
|  |  | Reverse | AAACGACCAAACGAGAAACGAA |
| *asdA* | N646_0788 | Forward | GCAACAGGCTGGCGTTTAGG |
|  |  | Reverse | GCTGTCTGCTACGATACGGTCAAT |
| *bioF* | N646_0165 | Forward | AGGGTTGAATCGTTCTATGAATGTG |
|  |  | Reverse | CGGTCAGTGCGGCTTCTAAAT |
| *alaS* | N646_1642 | Forward | CCAACCCTGCTGTTCACAAAC |
|  |  | Reverse | CAACGCTGAGCCGTAGTCG |
| *alaS* | N646_2770 | Forward | AGGTTGGGTGTTTGTCGTCG |
|  |  | Reverse | GTCTTTGCGGTCTGCGTCTT |
| *murC* | N646_2616 | Forward | GCTGTTGAACGAAGGCTACCA |
|  |  | Reverse | GACCAATAAATACCGTCGCACC |
| *alr* | N646_1828 | Forward | ACCTATTGCAGGTCGAGTTTCC |
|  |  | Reverse | TCGCTTCGTCGCTGACTTTAT |
| *alr* | N646_4376 | Forward | ATACGGAGACTTACCAACTAACCCTG |
|  |  | Reverse | TGAAGCAACGCCCGCAAC |
| *avtA* | N646_2197 | Forward | GGCAGGTAAACAGCCAGACG |
|  |  | Reverse | GGGTTAGTTGGGCGAGACG |
| *alaA* | N646_3077 | Forward | TATGTAGGTAACGGCGTGTCTGA |
|  |  | Reverse | CGGATAGTCTGGTGCTGGGAT |
| *ald* | N646_0156 | Forward | CCTTGTTATCGGTGCCGTTCT |
|  |  | Reverse | TTGGATCTGCGTGAGTTGTCG |
| *phnW* | N646_3669 | Forward | ATTACTAGAGCTGGAACAAGAAGGC |
|  |  | Reverse | TGATGATTGGCGAGTGAAGGT |
| *pucG* | N646_1805 | Forward | CGTCTGCCGTAATGGTGTATTTG |
|  |  | Reverse | TGTATCTGGGTGCTGTTCGAGTG |
| *crp* | N646_1044 | Forward | GCTCAAGGTGCAGTGAGAGT |
|  |  | Reverse | ACCTTTTGGCGACTCGTTCT |
| *cyaA* | N646_2038 | Forward | ACTCGTTTACATGCCGTGGT |
|  |  | Reverse | CTGCATAGATGGACGACGCT |
| *atpA* | N646_2171 | Forward | TGACCTTGATGACGCAACGA |
|  |  | Reverse | TCGTTGTAAGCGCCAGACTT |
| *atpB* | N646_2175 | Forward | GGCTATGGCTCTAGGCGTTT |
|  |  | Reverse | CCACGGTAGTGAACCCATCC |
| *atpC* | N646_1182 | Forward | ATTCAGCCTGGTACAGCGAC |
|  |  | Reverse | GCTTGTGCGAAGTCCATGTC |
| *atpD* | N646_2169 | Forward | CGTAACATCGCTGCAATGGG |
|  |  | Reverse | AGTCTGCTGAACACCACGAG |
| *atpE* | N646_2174 | Forward | TTTCTGCAATCGCCGTAGGT |
|  |  | Reverse | ACCGATCATTGGAACCGCAT |
| *atpF* | N646_2173 | Forward | TTGAAAGAAGCGAAGCGCAC |
|  |  | Reverse | TAGCAACTTGTTTGCGCAGC |
| *atpG* | N646_2170 | Forward | GCTTCTCGTCCATACGCTGA |
|  |  | Reverse | AGACCACGGTCTGTCGAAAC |
| *atpH* | N646_2172 | Forward | TTGACTACTATCGCACGCCC |
|  |  | Reverse | CATCAACTTGTTCGCCGCAA |
| *mnhD* | N646_4012 | Forward | AATTTGGGTCGCAGGGCTAA |
|  |  | Reverse | TGTAGAAAATGGCACCGGCT |
| *mnhF* | N646_4014 | Forward | CCAACGCTAGCTGATCGAGT |
|  |  | Reverse | CCGAGAAATGCGACCAAACC |
| *mnhG* | N646_4015 | Forward | AAGCTGGAACTGTCGGAGTG |
|  |  | Reverse | CTGGCGCAGTGAGAACGATA |
| *nhaB* | N646_1148 | Forward | TGGCGGTGGCAATCAATACT |
|  |  | Reverse | TCGCAAGAACGACGGTGTAA |
| *rnfE* | N646_1182 | Forward | TGCTGGGCTTATGTCCACTG |
|  |  | Reverse | ATTAATTCGCGCATGGCACC |
| *rnfG* | N646_1181 | Forward | CAACCGGATTAGTCGCCCTT |
|  |  | Reverse | TTCACCATTCTTCGTGGCGA |
